# Cranial Nerve Palsy and Risk of Kidney Cancer: A Nationwide Population-Based Study

**DOI:** 10.3390/medicina60060913

**Published:** 2024-05-30

**Authors:** Dongyoung Lee, Kyungdo Han, Soolienah Rhiu, Jin-hyung Jung, Kyung-Ah Park, Sei Yeul Oh

**Affiliations:** 1 Department of Ophthalmology, Samsung Medical Center, Sungkyunkwan University School of Medicine, Seoul 06351, Republic of Korea; pizza0324@gmail.com (D.L.);; 2 Department of Statistics and Actuarial Science, Soongsil University, Seoul 06978, Republic of Korea; 3 Department of Ophthalmology, Dongtan Sacred Heart Hospital, Hallym University School of Medicine, Hwaseong 18450, Republic of Korea

**Keywords:** kidney cancer, ocular motor cranial nerve palsy, sixth cranial nerve palsy, fourth cranial nerve palsy, third cranial nerve palsy

## Abstract

*Background and Objective:* Understanding whether cranial nerve palsy (CNP) acts as an independent risk factor for kidney cancer could have important implications for patient care, early detection, and potentially the development of preventive strategies for this type of cancer in individuals with CNP. This study aimed to examine the risk of kidney cancer following the onset of ocular motor CNP and assess whether CNP could be considered an independent risk factor for kidney cancer. *Materials and Methods:* A population-based cohort study was conducted using data from the National Sample Cohort (NSC) database of Korea’s National Health Insurance Service which was collected from 2010 to 2017. Follow-up was until kidney cancer development, death, or 31 December 2018. Cox proportional hazard regression analysis was performed to determine hazard ratios (HRs) for kidney cancer according to CNP status. Participants aged 20 years or more diagnosed with CNP from 2010 to 2017 were included. Exclusions comprised individuals with specific pre-existing conditions, inability to match a control group, and missing data, among others. CNP patients were age–sex matched in a 1:5 ratio with control cases. The primary outcome was incidence of kidney cancer during the follow-up period. *Results:* This study comprised 118,686 participants: 19,781 in the CNP group, and 98,905 in the control group. Compared to the control group, participants with CNP had a higher risk of kidney cancer (adjusted HR in model 4, 1.599 [95% CI, 1.116–2.29]). After a 3-year lag period, the CNP group had a significantly higher risk (adjusted HR in model 4, 1.987 [95% CI, 1.252–3.154]). *Conclusions:* Ocular motor CNP may be an independent risk factor for kidney cancer, as indicated by a higher incidence of kidney cancer in CNP patients. Further research is needed to elucidate the underlying mechanisms and explore potential preventive measures for kidney cancer in patients with ocular motor CNP.

## 1. Introduction

Ocular motor cranial nerve palsy (CNP), a condition that impairs the functioning of one or more of the cranial nerves governing ocular movement, is frequently encountered in neuro-ophthalmology practice [1,2,3,4,5], especially among the elderly population. This cranial nerve palsy results in ocular deviation, diplopia, abnormal head positioning, and various related symptoms depending on the etiology and the location of the lesion [2,6,7,8]. The condition can be caused by various factors, including head trauma, infections, tumors, and vascular disorders, and the main etiology is regarded as an atherosclerotic condition affecting small blood vessels [1,3,4,5,9]. Although ocular motor CNP itself is not usually considered a life-threatening condition, recent research has suggested a potential link between this condition and an increased risk of serious medical problems such as ischemic stroke [10,11,12]. Several studies have demonstrated an association between various metabolic risk factors, such as diabetes, hyperlipidemia, and obesity, and the development of renal cell carcinoma (RCC), the most common type of kidney cancer [13,14,15]. It is proposed that this association between these metabolic risk factors and kidney cancer may be a result of adipose dysfunction leading to the abnormal secretion of cytokines and the activation of insulin-like growth factor (IGF)-I, thereby promoting cellular proliferation and oxidative stress, factors conducive to kidney cancer development [13,14,15]. Given the shared common risk factors between these two medical conditions, it would be meaningful to investigate the potential relationship between ocular motor CNP and kidney cancer. The purpose of this study was to investigate the risk of kidney cancer subsequent to the onset of ocular motor CNP and to determine whether CNP serves as an independent risk factor for kidney cancer after adjusting for potential compounding factors. This was achieved using the National Sample Cohort (NSC) database from Korea’s National Health Insurance Service (KNHIS) with a specific focus on the adult Korean population.

## 2. Materials and Methods

### 2.1. Data Sources and Study Population

The data we used were obtained from Korea’s National Health Insurance (NHI), which are anonymized data including almost 97% of the whole Korean population and publicly available to all researchers who are approved by the Institutional Review Board (IRB). Codes for diagnosis were based on the International Classification of Diseases-10 (10th revision).

**Figure 1:**
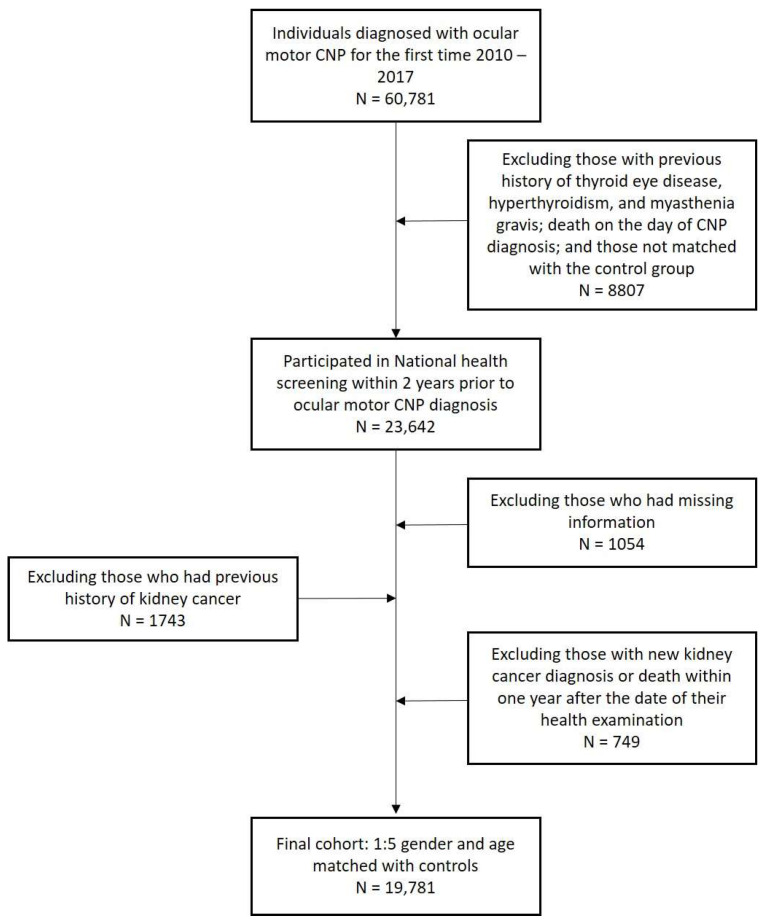
Flowchart of participant inclusion and exclusion. CNP: cranial nerve palsy.

In this study, individuals aged 20 years or more who underwent KNHIS health examinations and were diagnosed as ICD-10 code of CNP (H49.0, H49.1, H49.2) from 1 January 2010 to 31 December 2017 were analyzed. A total of 60,781 CNP cases were diagnosed during this period. Study participants were followed up until the development of kidney cancer or death, or until 31 December 2018. The inclusion and exclusion criteria are shown in Table 1. Figure 1 shows the flow diagram of participant selection. We excluded participants who were diagnosed with dysthyroid exophthalmos, thyrotoxicosis, or myasthenia gravis before the diagnosis of CNP (*n* = 52,076); those who were unable to match with a control group (*n* = 51,975); those who died on the day of the diagnosis of CNP (*n* = 51,974); those who did not undergo a national health screening program within 2 years before the diagnosis of CNP (*n* = 23,642); individuals with missing health examination data (*n* = 22,588); those with any cancer history before cohort entry (20,845); and those who were newly diagnosed with any type of cancer within one year of their diagnosis with CNP (20,096). The reason we set the 1-year time lag was to avoid a situation where the causal relationship was reversed. After excluding these cases, 20,096 participants with CNP were left, and after age–sex matching in a 1:5 ratio with control cases, 19,781 CNP cases were included with an additional 98,905 control cases. Health examination data included smoking status, height, weight, body mass index (BMI), waist circumference, serum glucose level, cholesterol level, systolic blood pressure (SBP), and diastolic blood pressure (DBP).

The study adhered to the tenets of the Declaration of Helsinki and was approved by the Institutional Review Board (IRB) of Samsung Medical Center (IRB no. 2023-05-099).

### 2.2. Definitions of Kidney Cancer, Ocular Motor CNP, and Confounders

The study endpoint was kidney cancer occurrence from the index date up until the end of the year 2015. The confirmation of incident kidney cancer was made using the ICD-10-CM code C64 in conjunction with the registration code V193.

Ocular motor CNP was defined by the ICD-10 codes H49.0 (3rd CNP), H49.1 (4th CNP), or H49.2 (6th CNP). The ICD-10 codes of H06.2 (dysthyroid exophthalmos), E05 (thyrotoxicosis), or G70.0 (myasthenia gravis) were excluded. Variables like sex, age, smoking status, amount of alcohol consumption, regular exercise, income status, and obesity were considered as confounders. According to Mesh definitions, sex was stratified as male/female, as Korea’s social security numbers (SSNs) that start with 1 represent males and 2 females, and participants were automatically categorized by their SSN into the KNHIS.

Regarding smoking status, participants were divided into smokers versus non-smokers. Drinking was classified into non-drinkers, mild drinkers, and heavy drinkers according to the amount of alcohol consumption. We defined mild drinkers as those who consumed less than 30 g of alcohol and heavy drinkers as those who consumed 30 g or more. Regular exercise was defined as performing ≥20 min of vigorous-intensity physical activity at least 3 times per week or ≥30 min of moderate-intensity physical activity at least 5 times a week. Income status was divided into 4 quartiles (Q), with Q1 indicating the lowest and Q4 indicating the highest income. Obesity was defined as BMI ≥ 25 kg/m^2^ based on World Health Organization (WHO) recommendations for Asian populations.

Comorbidities like diabetes mellitus, hypertension, dyslipidemia, and chronic kidney disease were also included and adjusted in the analysis. Diabetes mellitus (DM) was defined as fasting glucose ≥126 mg/dL or was identified by using diagnostic codes E10-14. Hypertension (HTN) was defined when BP ≥ 140/90 mmHg or ICD-10-CM codes with a prescription for antihypertensive medications were present. Dyslipidemia was defined as serum total cholesterol levels ≥240 mg/dL or with the ICD-10-CM code E78 with the use of a lipid-lowering drug. Chronic kidney disease (CKD) was defined as an estimated glomerular filtration rate < 60 mL/min per 1.73 m^2^.

### 2.3. Statistical Analysis

Baseline characteristics were compared using Student’s *t*-test for continuous variables and the chi-square test for categorical variables. Data were mainly presented as a number of participants or mean ± SD. For the adjustment of confounders, four models were set and compared independently. Model 1 was analyzed without adjusting factors, while model 2 was adjusted for sex and age as confounders. Model 3 adjusted for variables including smoking, drinking amount, regularity of exercise, income status, and obesity in addition to model 2 confounders. Lastly, model 4 adjusted for DM, HTN, dyslipidemia, and CKD in addition to model 3 confounders. A Cox proportional hazard regression analysis was conducted to determine hazard ratios (HRs) and 95% CIs for kidney cancer according to CNP. All data analyses were performed using SAS version 9.4 (SAS Institute Inc., Cary, NC, USA), and a *p*-value < 0.05 was considered statistically significant.

## 3. Results

### 3.1. Baseline Characteristics of the Study Population

The general characteristics of the study population are shown in Table 2. Of the 118,686 participants in our study, 98,905 individuals were in the control group, and 19,781 individuals were in the CNP group. Compared with the control group, participants with CNP showed lower alcohol consumption; a lower percentage of Q1 income status; an increased likelihood of obesity, DM, HTN, dyslipidemia, and CKD; higher weight, BMI, waist circumference, glucose level, SBP, and DBP; and lower cholesterol levels.

### 3.2. Risk for Kidney Cancer According to CNP

In our study group, a total of 165 participants were diagnosed with kidney cancer during the follow-up period. Compared with the control groups, participants with CNP were at increased risk of kidney cancer (incidence rate, 0.473 versus 0.284 cases per 1000 person-years, respectively; adjusted HR in model 4, 1.599 [95% CI, 1.116–2.29]). Moreover, with a 3-year lag period, the CNP group had a higher risk of kidney cancer than the control group (incidence rate, 0.529 versus 0.265 cases per 1000 person-years, respectively; adjusted HR in model 4, 1.987 [95% CI, 1.252–3.154]) (Table 3).

**Figure 2:**
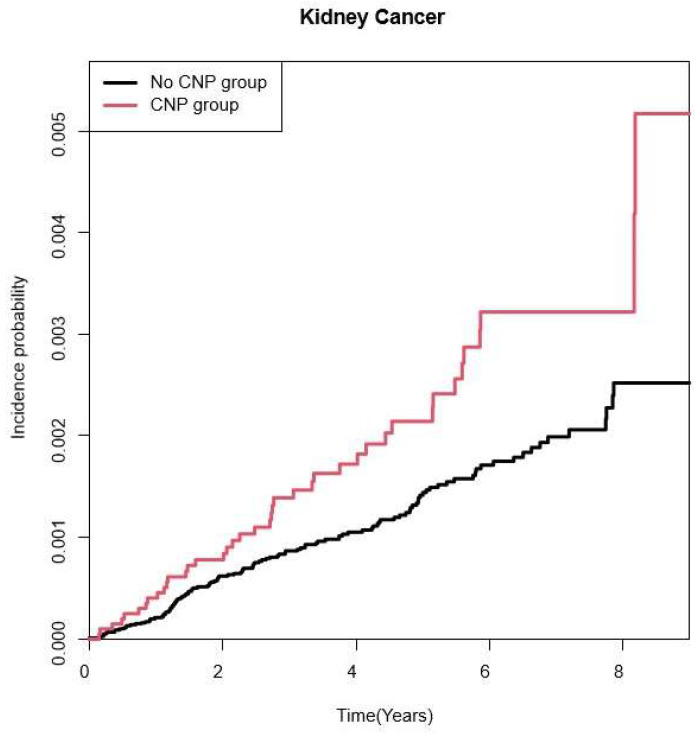
Kaplan–Meier curve for cumulative incidence probability of kidney cancer with the diagnosis of cranial nerve palsy (*p* of log-rank = 0.0041). CNP: cranial nerve palsy.

The Kaplan–Meier curve for the incidence probability of kidney cancer with CNP is shown in Figure 2, indicating that the CNP group had more incidence probability of kidney cancer (*p*-value = 0.004).

### 3.3. Subgroup Analysis

In Table 4, the relative risks of kidney cancer stratified by sex, age, smoking, alcohol consumption, regular exercise, income status, obesity, DM, HTN, dyslipidemia, and CKD are shown. All subgroups showed no significantly different associations between CNP and the risk of kidney cancer, but the amount of alcohol consumption showed a marginal difference in that non-alcohol drinkers had a higher risk for kidney cancer.

## 4. Discussion

Acquired CNP can be attributed to various etiologies, including vascular diseases, trauma, neoplasms, aneurysms, and other miscellaneous causes [16,17]. An earlier investigation into a Korean cohort revealed that neoplasm being the cause of CNP included multiple neuromas in neurofibromatosis type 2, brain stem glioma, juxtasellar meningioma, intracranial invasion of nasopharyngeal cancer, and posterior fossa epidermoid cysts [16]. However, to the authors’ knowledge, there is no study analyzing the risk of new-onset cancer following CNP. This study is the first report to show that CNP patients have an increased rate of kidney cancer. An essential aspect of our study design was the incorporation of a 3-year lag period to minimize the risk of reverse causation, where the undiagnosed metastatic kidney cancer, which caused CNP, could be misinterpreted as occurring after the onset of CNP. Importantly, when we implemented this 3-year lag period, the association between CNP and kidney cancer risk was found to be stronger than with the 1-year lag period. This higher correlation further supports the hypothesized relationship between CNP and kidney cancer, suggesting that a prolonged duration between the occurrence of CNP and the detection of kidney cancer intensifies their interrelationship.

Kidney cancer ranks as the 12th most common malignant neoplasm, excluding blood cancers, and accounts for about 2.4% of all malignancies in adults, and the incidence has been increasing world-wide [18,19]. Based on 2020 estimates from GLOBOCAN data, North America had the highest incidence of age-standardized rate (ASR) at 12.2, followed by Australia and New Zealand at 10.0 and Europe at 9.5 [18,20]. Although ASRs in Asia were low at 2.8, those in Japan and South Korea were high at 7.6 and 6.5, suggesting that not only race but also lifestyle plays a role in their incidence [18,20].

Smoking, hypertension, and obesity are well-established risk factors for kidney cancer [13,14,15,18,19]. Tobacco smoke contains a mixture of carcinogens implicated in the etiology of kidney cancer, and there is a dose–response relationship between risk and the amount smoked per day [21,22]. Hypertension is independently associated with kidney cancer risk [22] and evidence has been reported that hypertension predisposes kidney cancer [18]. The biological mechanism underlying the relationship between hypertension and kidney cancer remains unclear, but is hypothesized to involve chronic renal hypoxia and lipid peroxidation due to the formation of reactive oxygen species [18,23,24].

Ocular motor CNP also has a close association with vasculopathic risk factors such as diabetes mellitus, hyperlipidemia, and hypertension, suggesting microvascular ischemia to be the main etiology of CNP [25,26,27]. Although the mechanism between CNP and kidney cancer needs further studies, we postulate that microvascular ischemia may contribute to chronic renal stress leading to kidney cancer.

This study has several limitations that need to be acknowledged. First, due to the retrospective nature of the study design, it was impossible to confirm a causal relationship between ocular motor CNP and kidney cancer, and we were only able to demonstrate an association. Second, our findings are based on data from Korea’s National Health Insurance Service, which may not be generalizable to populations in other countries with different health systems, lifestyle factors, and genetic backgrounds. More multicentric and multinational studies are needed to validate our findings and to ensure their generalizability. Third, this study relied on diagnostic codes to identify CNP and kidney cancer cases, and the potential for misclassification bias exists. Despite the accuracy of these codes, the potential for coding errors cannot be entirely ruled out. Furthermore, we were unable to evaluate the severity, specific subtype, or treatment details of both CNP and kidney cancer, which could influence the outcomes. Lastly, despite adjusting for multiple confounders, potential residual confounding from unmeasured or unknown factors, such as a family history of kidney cancer, environmental exposures, dietary factors, and other potential risk factors for kidney cancer, cannot be eliminated. This study also did not account for changes in lifestyle or medical interventions over time that may have affected the risk of kidney cancer. Therefore, further prospective studies are required to confirm these findings and to elucidate the underlying mechanisms behind this association.

In conclusion, this large-scale, retrospective study highlights a previously unreported association between ocular motor CNP and an increased risk of kidney cancer. The strength of this association was particularly amplified when a 3-year lag period was applied, thereby underlining the relevance of a temporal relationship between CNP and kidney cancer.

Importantly, our findings extend the current understanding of the potential complications associated with CNP, suggesting a broader systemic involvement beyond the ocular system. Clinicians should consider these findings when managing patients with CNP, as the monitoring and early detection of kidney cancer could be critical for such patients. However, given the observational nature of our study, future prospective research is required to confirm these associations and explore the underlying pathophysiological mechanisms. This work opens a new avenue for understanding the systemic implications of CNP and offers a foundation for further studies aiming at early detection and preventive strategies for kidney cancer in CNP patients.

## Figures and Tables

**Table 1:** Inclusion and exclusion criteria of participants.

Criteria	
Inclusion	Aged 20 years or more and underwent KNHIS health examinations
	Diagnosed with ICD-10 code of CNP (H49.0, H49.1, H49.2) from 1 January 2010 to 31 December 2017
Exclusion	Dysthyroid exophthalmos
	Thyrotoxicosis
	Myasthenia gravis
	Unmatchable to control group
	Died on the day of the diagnosis of CNP
	Did not undergo national health screening program within 2 years before diagnosis of CNP
	Missing health examination data
	Cancer history before cohort entry
	Newly diagnosed cancer within one year since diagnosis of CNP

KNHIS: Korea’s National Health Insurance System, ICD: International of Classification of Diseases, CNP: cranial nerve palsy.

**Table 2:** Baseline characteristics of patients with ocular motor cranial nerve palsy and control patients.

Variables	Total	CNP		
		No	Yes	*p*-Value
	118,686	98,905	19,781	
Sex, male, *n* (%)	74,778 (63)	62,315 (63)	12,463 (63)	1
Age, years	59.82 ± 13.05	59.82 ± 13.05	59.82 ± 13.05	1
20–39	9282 (7.82)	7735 (7.82)	1547 (7.82)	1
40–64	62,010 (52.25)	51,675 (52.25)	10,335(52.25)	
≥65	47,394 (39.93)	39,495 (39.93)	7899 (39.93)	
Income status Q1, *n* (%)	23,973 (20.2)	20,126 (20.35)	3847 (19.45)	0.004
Smoking status, smoker, *n* (%)	26,318 (22.17)	22,014 (22.26)	4304 (21.76)	0.1227
Drinking amount, *n* (%)				
None	67,224 (56.64)	55,454 (56.07)	11,770 (59.5)	<0.0001
Mild	42,361 (35.69)	35,736 (36.13)	6625 (33.49)	
Heavy	9101 (7.67)	7715 (7.8)	1386 (7.01)	
Regular exercise, *n* (%)	25,379 (21.38)	21,104 (21.34)	4275 (21.61)	0.3909
Obesity, *n* (%)	44,288 (37.32)	36,386 (36.79)	7902 (39.95)	<0.0001
DM, *n* (%)	21,978 (18.52)	16,029 (16.21)	5949 (30.07)	<0.0001
HTN, *n* (%)	53,620 (45.18)	43,508 (43.99)	10,112 (51.12)	<0.0001
Dyslipidemia, *n* (%)	39,819 (33.55)	31,729 (32.08)	8090 (40.9)	<0.0001
Chronic kidney disease	8970 (7.56)	7350 (7.43)	1620 (8.19)	0.0002
Height, cm	162.77 ± 9.1	162.75 ± 9.11	162.86 ± 9.05	0.1213
Weight, kg	64.15 ± 11.18	64.02 ± 11.11	64.81 ± 11.48	<0.0001
BMI, kg/m^2^	24.13 ± 3.15	24.09 ± 3.13	24.35 ± 3.24	<0.0001
Waist circumference (cm)	82.83 ± 8.83	82.69 ± 8.78	83.56 ± 9.03	<0.0001
Fasting glucose (mg/dL)	104.34 ± 29.94	102.75 ± 26.68	112.29 ± 41.74	<0.0001
Systolic BP (mmHg)	125.77 ± 15.33	125.64 ± 15.26	126.43 ± 15.64	<0.0001
Diastolic BP (mmHg)	77.29 ± 9.97	77.23 ± 9.91	77.6 ± 10.24	<0.0001
Total cholesterol (mg/dL)	195.22 ± 38.36	195.52 ± 38.07	193.71 ± 39.77	<0.0001
eGFR (mL/min/1.73 m^2^)	87.78 ± 43.26	87.69 ± 41.6	88.2 ± 50.73	0.1326
Renal cancer	165 (0.14)	124 (0.13)	41 (0.21)	0.0048
F/U duration				
Mean ± SD	4.42 ± 2.24	4.42 ± 2.24	4.38 ± 2.24	0.0345
Median (Q1–Q3)	4.19 (2.46–6.24)	4.2 (2.47–6.24)	4.16 (2.43–6.2)	0.0706

CNP: cranial nerve palsy; Q1, 2, and 3: the 1st, 2nd, and 3rd quartiles; DM: diabetes mellitus; HTN: hypertension; BMI: body mass index; BP: blood pressure; eGFR: estimated glomerular filtration rate; F/U: follow-up; SD: standard deviation.

**Table 3:** Subgroup analysis of the incidence of ocular motor cranial nerve palsy according to the time lags of 1 year and 3 years.

	CNP	*n*	Renal Cancer	Duration	Incidence Rate (Per 1000 PY)				
						Model 1	Model 2	Model 3	Model 4
Time lag of 1 year	No	98,905	124	437,287.18	0.284	1 (Ref.)	1 (Ref.)	1 (Ref.)	1 (Ref.)
	Yes	19,781	41	86,727.79	0.473	1.667 (1.171, 2.373)	1.667 (1.171, 2.373)	1.652 (1.16, 2.353)	1.599 (1.116, 2.29)
Time lag of3 years	No	81,330	66	248,929.62	0.265	1 (Ref.)	1 (Ref.)	1 (Ref.)	1 (Ref.)
	Yes	16,179	26	49,142.39	0.529	1.996 (1.268, 3.142)	1.997 (1.268, 3.144)	1.982 (1.258, 3.123)	1.987 (1.252, 3.154)

CNP: cranial nerve palsy; *n*: numbers; PY: person-years. Model 1 was analyzed without adjustment for confounders. Model 2 adjusted age and sex as confounders. Model 3 adjusted age, sex, smoking status, drinking amount, regularity of exercise, status of income, and obesity. Model 4 adjusted age, sex, smoking status, drinking amount, regularity of exercise, status of income, obesity, and comorbidities including hypertension, diabetes mellitus, dyslipidemia, and chronic kidney disease.

**Table 4:** Subgroup analysis of the association between cranial nerve palsy and kidney cancer in the adjusted Cox model.

Subgroup		HR (95% CI)	*p* for Interaction
Sex	Male	1.418 (0.938, 2.143)	0.2012
	Female	2.437 (1.18, 5.034)	
Age	20–39	.	.
	40–64	1.665 (0.997, 2.783)	
	65–	1.672 (1.014, 2.757)	
Smoking	No	1.642 (1.093, 2.468)	0.7892
	Yes	1.464 (0.696, 3.078)	
Drinking amount	No	2.108 (1.349, 3.294)	0.1301
	Mild	1.239 (0.64, 2.4)	
	Heavy	0.359 (0.048, 2.707)	
Regular exercise	No	1.527 (1.015, 2.298)	0.6297
	Yes	1.876 (0.898, 3.919)	
Income status	Other	1.542 (1.036, 2.296)	0.6679
	Low Q1	1.878 (0.833, 4.232)	
Obesity	No	1.767 (1.107, 2.822)	0.5269
	Yes	1.403 (0.81, 2.429)	
DM	No	1.487 (0.951, 2.324)	0.5751
	Yes	1.848 (0.997, 3.426)	
HTN	No	1.822 (1.037, 3.201)	0.5648
	Yes	1.473 (0.93, 2.333)	
Dyslipidemia	No	1.524 (0.959, 2.424)	0.7442
	Yes	1.72 (0.98, 3.018)	
Chronic kidney disease	No	1.456 (0.976, 2.171)	0.2417
	Yes	2.503 (1.102, 5.686)	

HR: hazard ratio; CI: confidence interval; Q1: the 1st quartile; DM: diabetes mellitus; HTN: hypertension.

## Data Availability

The data presented in this study are available on request from the corresponding author due to legal issues.
